# A novel psychometric approach to assessing intersectional hiv stigma: the geometric intersectional stigma scales

**DOI:** 10.1007/s10865-022-00331-4

**Published:** 2022-06-10

**Authors:** Seth C. Kalichman, Bruno Shkembi, Lisa A. Eaton

**Affiliations:** Institute for Collaboration on Health Intervention and Policy, University of Connecticut, Connecticut, United States

**Keywords:** Intersectional stigma, HIV stigma, HIV/AIDS, Health behaviors

## Abstract

Recent advances in stigma theories have emphasized intersectionality, but there are currently few approaches to measuring intersectional HIV stigma. Here we present a novel approach to assessing intersectional HIV stigma. Black/African American sexual minority men living with HIV (N = 437) completed newly developed measures of enacted and anticipated stigma along with a battery of instruments to examine construct validity. For each endorsed stigma item, participants rated three personal attributes ascribed to the stigma experiences, specifically race, same-sex sexual behavior, and HIV status. Based on the notion that intersecting orthogonal dimensions such as attributions for experiencing stigma can be conceptualized geometrically, we used the Pythagorean Theorem to calculate intersectional stigma scores. Results showed that the enacted and anticipated stigma scales and the assessment of intersectional stigma attributes demonstrated acceptable response rates, internal consistency and a pattern of associations with correlates that suggests construct validity. In separate tests of construct validity, regression models predicting medication adherence indicated varied results among stigma measures, demonstrating clear advantages to separating the assessment of stigma experiences from the attributes to which stigma experiences are ascribed. Findings from this geometric approach to assessing intersectional HIV stigma were promising and warrant further investigation.

## Introduction

Stigma is inextricably entwined with the HIV epidemic itself (Herek & Glunt, 1988). In part, stigma may function as a socially protective process (Pirlott & Cook, 2018), distancing individuals from the perceived threat of pathogens in a behavioral immune system (Kusche & Barker, 2019). Stigma is experienced by people living with HIV (PLWH) through three distinct mechanisms; Enacted stigma – acts of prejudice, stereotyping, and discrimination by others against PLWH; Anticipated stigma - expecting to experience prejudice, stereotyping, and discrimination in the future; and Internalized stigma – personally adopting negative beliefs and feelings associated with societal stigma (Earnshaw & Chaudoir, 2009; Turan et al., 2017). All three stigma mechanisms are known to impede progression along the HIV continuum of care, including antiretroviral therapy (ART) adherence and viral suppression (Herek & Capitanio, 1993; Herek & Glunt, 1988; Kalichman & Simbayi, 2003; Kalichman & L. C. Simbayi, 2003; Kelly et al., 2016; Treves-Kagan et al., 2017; Turan et al., 2011).

Along with stigma ascribed to HIV are stigmas attached to modes of HIV transmission (e.g., same-sex sexual behavior, injection drug use), populations most affected by HIV (e.g., racial and sexual minorities), and contextual factors (poverty, non-injection drug use), with the various stigmatized attributes intersecting to form complex and unique life experiences (Bowleg et al., 2017; Logie et al., 2011; Pachankis et al., 2017). Research has generally approached assessing intersectional stigma in three ways: (a) at the item level, referencing multiple personal attributes within each item such that individuals are asked to rate their experiences as a person of specified attributes [e.g., as Black man living with HIV who has male sex partners] (Jackson et al., 2020); (b) at the scale level, allowing individuals to ascribe the personal attributes they believe are the bases for their stigma experiences (Scheim & Bauer, 2019); and (c) statistically, using interaction terms and moderator analyses in a regression framework (Earnshaw et al., 2013). Each of these approaches has advantages and disadvantages. For example, at the item-level stigma experiences are keyed to the intersecting attributes, making it impossible to separate the stigma experiences from their attributions as well as impossible to know whether stigma was attributed to one attribute or another, or both attributes as intended (Turan et al., 2019).

**Fig. 1:**
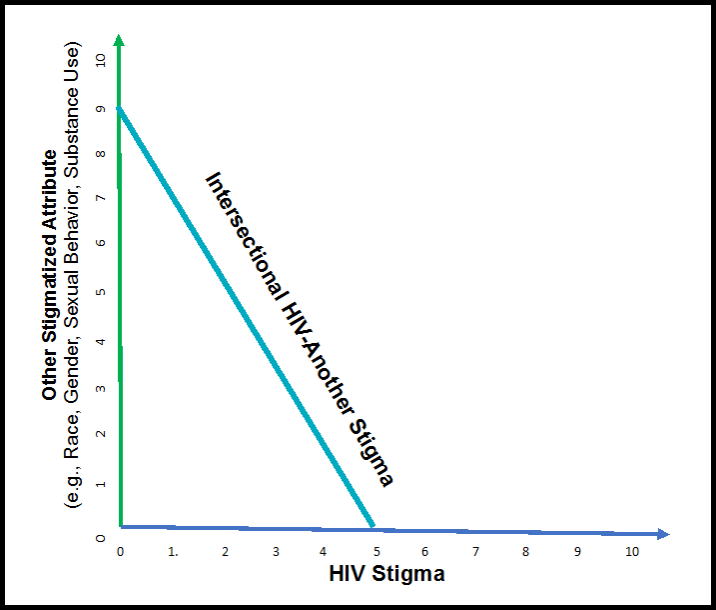
Geometric conceptualization for developing an intersectional stigma scale

Philosophical perspectives of intersectionality have also guided the conceptualization of intersectional stigma (Jackson-Best & Edwards, 2018; Mena et al., 2019; Crenshaw, 1989; Weldon, 2008), for example, have discussed intersectionality as the interaction between two or more ‘axes of domination’ and that the ‘axes of social relations’ do not operate autonomously. A novel approach to measuring intersectional stigma has recently been proposed in which the notion that intersectionality, conceptualized as a nexus of axes, is taken in a literal sense by implementing a geometrical definition of intersection; the point or points linking straight lines (Kalichman et al., 2021). As shown in Fig. 1, intersectional stigma scores are based on intersecting personal attributes that an individual ascribes to their stigma experiences by using the Pythagorean Theorem, c = $\surd$ (a^2^ + b^2^) and its extension to multiple attributes d = $\surd$ (a^2^ + b^2^ + c^2^). In this approach, HIV stigma frameworks (Earnshaw & Chaudoir, 2009; Turan et al., 2019) informs the selection and adaptation of items to assess enacted and anticipated stigma. In addition, theories of intersectionality (Crenshaw, 1989; Weldon, 2008) form the basis for creating intersectional stigma scores. A geometric approach to measuring intersectional stigma therefore incorporates elements from item-level intersectional stigma scales by asking respondents if they have had or expect to have specific stigma experiences within each item (e.g., item-level). It also draws on scale-level methods by obtaining independent ratings to ascribe the stigma experience to personal attributes and uses a statistical approach to mathematically calculate intersectional stigma scores.

An example may help to illustrate the geometric approach to assessing intersectional stigma. Respondents are first asked about their stigma experiences, such as whether they have been socially isolated or discriminated against, and then they rate the extent to which they experienced (or anticipate experiencing) the event because of personal attributes. For example, they may rate each stigma experience as having occurred because of (a) their race, (b) same-sex sexual behavior, and/or (c) HIV status. Each attribution is independently rated on 10-point scales, such that zero means that they do not perceive the attribute as a contributing factor and 9 means they believe it did occur because of the attribute. Consider a sexual minority man of color who is living with HIV who says he has experienced exclusion from social situations and attributes the experience to his race (score 9), but not at all to his HIV status (score 0). In this case, his race-HIV intersectional stigma score would be 9 = $\surd$ (9^2^ + 0). Alternatively, let’s say that he attributes being excluded from social situations to both his race with a rating of 9 and his HIV status with a rating of 5. In this case the intersectional race-HIV stigma score will be 10.3 = $\surd$ (9^2^ + 5^2^). Thus, in this example 4 different stigma scores are obtained - the stigma item endorsed, the ratings ascribing the stigma to two attributes (race and HIV status), and the race-HIV status intersectional stigma score.

In the current study, we examined the functional properties of the newly developed enacted and anticipated HIV stigma scales in a sample of Black/African American sexual minority men living with HIV. We also examined potential advantages of separating the stigma experiences from the intersecting attributes by testing the associations of the enacted and anticipated intersectional stigma scales in relation to a battery of potential correlates, including ART adherence.

## Methods

### Participants

A total of 1,874 individuals were screened for participation, of which 815 (43%) responded to Facebook announcements and the remaining 1,059 had heard about the study from previous participants or having participated in previous studies. A total of 1,130 (60%) were either HIV negative or unknown HIV status or did not identify as Black/African American. Among the 744 HIV positive persons, 533 (72%) identified as men who have had sex with men (i.e., sexual minority men). A total of 437 Black/African American cisgender sexual minority men living with HIV completed surveys.

### Procedures

Participants were recruited through social media platforms (Facebook) and ‘word of mouth’. Potential participants completed an online form that indicated their interest in the study and were contacted by study staff for a brief ‘face-to-face’ video chat interview. This procedure was used to assure that all participants personally identified as Black men and showed documentation of their HIV status, such as an ART bottle, viral load lab result etc. with their name matched to a photo ID that showed their age was 18 or older. Eligible participants were sent a link to complete the measures using REDCap (Research Electronic Data Capture) web-based surveys. Participants received a $25 gift card for completing the stigma measures, after which they were sent a link to a second survey that included a battery of other measures for which they also received $25 for completing. The median time between completing the first survey and starting the second survey was 23 min. Data were collected between April, 2020 and November, 2020. All procedures were approved by the University of Connecticut institutional review board.

### Measures

*Enacted Stigma*. Participants responded to 24 items adapted from measures of enacted stigma, microaggressions, and discrimination scales (Balsam et al., 2011; Earnshaw & Chaudoir, 2009; Eaton et al., 2020; Genberg et al., 2008). As described elsewhere (Kalichman et al., 2021), items were rationally selected on the basis of contemporary theories of stigma to reflect discrimination, interpersonal stigma and felt stigma experiences (Major et al., 2018). The 24 enacted stigma items were responded to using 4-point scales, from 0 = ‘Never experienced’, to 3 = ‘Often experienced’. Following responding to each enacted stigma item, participants were asked to rate their perceived reasons for the experiences on four attributes: race, same-sex sexual behavior, HIV status, and other reasons. Participants also rated how distressing the experience was for them. Instructions for the enacted stigma items read “The following section describes negative experiences that people may have. For each, respond whether you have had the experience. If you have had the experience, tell us how upsetting it was for you by moving the slide-bar. Also tell us how much you think the experience had to do with your being Black/African American, your being a man who has had sex with other men, your being HIV positive, or some other reason. Move each of the slide-bars to show your answers.” The ratings for distress and ascribing the stigma experiences to attributes were placed on 100 point slide-bars that were anchored with a range from 0 = ‘Not at all’, to 100 = ‘Very much’.

*Anticipated Stigma*. Participants responded to 20 anticipated stigma items adapted from previous measures (Earnshaw & Chaudoir, 2009). Anticipated stigma items mirrored 20 of the 24 enacted stigma items described above, framing the items around the future and reflecting discrimination, interpersonal stigma, and felt stigma experiences. Instructions for the anticipated stigma scale read “The next statements are about experiences that you MAY have in the FUTURE. Select the answer that shows how likely or unlikely you think it is that these will happen to you in the FUTURE. For each, respond how likely you believe it is that you will have this experience. For those experiences you believe you may have in the future, tell us how much you think the experience would have to do with your being Black/African American, you’re being a man who has had sex with other men, your being HIV positive, or some other reasons.” Anticipated stigma items used slide bars anchored from 0 = ‘Extremely unlikely’, to 100 = ‘Extremely likely’.

*Calculating geometric Intersectional Stigma.* As mentioned above, for both the enacted and anticipated stigma scales, ratings for attributes ascribed to stigma experiences ranged from 0 to 100 points using slide bars. Intersectional stigma scores were calculated using the formula, c = $\surd$ (a^2^ + b^2^), where c is the intersectional score, and a and b are the ratings for two personal attributes. By extension, multiple stigmatized attributes were scored using the formula, d = $\surd$ (a^2^ + b^2^ + c^2^). Thus, in addition to the scale scores representing enacted and anticipated stigma, separate scores are calculated for each personal attribution and their intersections ascribed to the endorsed stigma experience. Because intersectional stigma scores ranged from 0 to 141 for two attributes, and 0 to 173 for three attributes, the intersectional scores were standardized to the same 0 to 100 point metric as the initial attribute ratings.

*Stigma correlates.* The following measures were included as correlates of stigma among people living with HIV. All measures were calculated as mean scores. *Stigma Distress.* As described above, each enacted stigma item that participants endorsed was rated with respect to ‘how upsetting’ the experiences were on 100 point slide-bars anchored with a range from 0 = ‘Not at all’, to 100 = ‘Very much’. Stigma distress scores were calculated by taking the mean rating across all endorsed enacted stigma items. *Depression symptoms*. The Centers for Epidemiological Studies Depression scale (CESD) was used to assess symptoms of depression (Radloff, 1977; Van Dam & Earleywine, 2011). The CESD is a widely used 20-item scale that assesses symptoms of depression in a 7-day timeframe, Cronbach’s alpha (α) = 0.89. *L*oneliness was assessed using an adapted version of the UCLA Loneliness Scale short-form (Russell, 1996). We used eight items that reflect social isolation and feelings of being alone that were internally consistent, α = 0.90. *Internalized HIV stigma* is defined by the adoption of social stigma beliefs by the stigmatized population and was measured using the 10-item *Internalized AIDS-Related Stigma Scale* (Kalichman et al., 2009), α = 0.92. *Stigma avoidance* was assessed with four specific behaviors intended to conceal HIV status responded to on 4-point scales, 0 = ‘Never’, to 3 = ‘Often’ (Kalichman et al., 2020). *Medical mistrust* was measured using eight items adapted from a group based medical mistrust scale developed for use with Black men (Shelton et al., 2010), α = 0.90. For ART adherence, we used the 3-item self-report instrument for retrospective adherence (IRA) developed and validated by Wilson et al., (2014). The adherence items represent the number of days medications were taken over the previous 30 days, the frequency of taking medications as directed, and a self-perception rating of how well medications were taken over the previous 30 days. Methods suggested by Wilson et al. were used to convert scores for each item on a scale of 0 to 100 using linear transformations and calculating the mean across the three items to a single adherence score with a range from 0 to 100, interpreted as percent adherence over the past 30 days, α = 0.73.

### Statistical analyses

Descriptive analyses were performed to examine item response frequencies and distress ratings for each of the enacted stigma items. Internal consistency was assessed with Cronbach’s alpha coefficients. In addition, we examined the means and standard deviations for the enacted and anticipated stigma scales and their associated attributes and intersectional attributes. Pearson correlation coefficients are reported for all intercorrelations within and between enacted and anticipated stigma scales and their respective attributes as well as for associations between stigma scores and the stigma correlates. Finally, we examined the associations between ART adherence and enacted and anticipated stigma attributes using multiple regression models controlling for the stigma scale scores. All statistical tests defined significance as p < .05.

## Results

The mean age of participants was 33.7 years (SD = 8.5, range 20–66). Two-thirds of participants (67%, n = 296) were unemployed and 33% (n = 145) had incomes under $10,000. All participants reported having at least one male sex partner in the previous 6-months, with 82% (n = 361), identifying as ‘gay/same gender loving’ and 5% (n = 21) identifying as ‘heterosexual/straight’.

### Frequencies of stigma experiences and associated stigma distress

Table 1 shows the frequencies of participant endorsement for the 24 enacted stigma items and their associated distress ratings arrayed from the most to the least frequently experienced. The most frequently experienced stigma experiences concerned interpersonal interactions, including feeling stereotyped and judged by others. The least frequently experienced stigma experiences concerned healthcare discrimination and being targeted by ‘bigots’. The most distressful stigma experiences were among the least frequently encountered, however all of the distress scores for stigma experiences were greater than the scale mid-point. The enacted stigma and anticipated stigma scales were internally consistent, both α’s = 0.96.

**Table 1:** Frequencies of experiencing enacted stigma items and mean distress ratings

	Endorsed		Distress Score	
	N	%	Mean	SD
I have heard people make jokes about people like me.	299	68	68.7	28.6
I have been called hurtful names and slurs.^a^	282	64	68.9	29.5
People have stereotyped me.^a^	276	63	62.9	30.0
I feel that others have judged me.^a^	274	62	62.0	28.8
People have made false assumptions about me.^a^	254	58	61.1	37.7
I have experienced prejudice.^a^	243	55	76.4	24.0
I have felt disrespected by people who do not even know me.	239	54	67.2	27.6
I have been treated unfairly.^a^	235	53	73.3	23.8
I have been unfairly rejected by potential dating or sexual partners.^a^	213	48	64.4	27.2
Others have acted uncomfortably around me.^a^	208	47	59.4	30.8
People who do not know me have expected me to act a certain way.^a^	202	46	51.0	31.5
There are times when I feel I am being treated unfairly by other people.^a^	192	43	66.6	26.3
I have been treated unfairly in stores or restaurants.^a^	176	40	75.4	24.3
I have felt invisible in the community.^a^	173	39	64.2	27.0
I have sensed that people who do not even know me have been uncomfortable around me.	170	38	58.5	30.4
I have been treated like less of a person.^a^	154	35	78.5	22.6
I have been treated as inferior by people that do not even know me.^a^	149	34	68.8	27.4
I have felt that I have not gotten a job or promotion because of who I am.	147	31	73.7	27.9
I have been unfairly excluded from social situations.^a^	128	29	64.0	25.8
I have been physically abused, such as being punched, hit, kicked, or beaten.^a^	113	25	87.9	17.6
Healthcare providers have not always given me the best treatment available.^a^	101	23	77.8	22.9
Healthcare providers have not always had my best interest in mind when treating me.^a^	99	22	77.5	25.1
I have been the target of bigots.^a^	100	22	74.8	26.2
I have been denied or not given proper medical care because of who I am.^a^	53	12	78.5	24.3

Note: Ordered by most to least frequently endorsed; ^a^ Items reframed for future tense and included in Anticipated Stigma Scale

### Intercorrelations within and between enacted and anticipated stigma scales

**Fig. 2:**
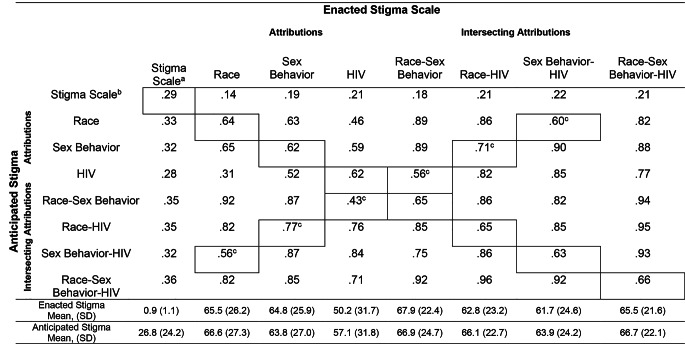
Inter-correlations among enacted (upper panel) and anticipated (lower panel) stigma and their respective attributes and geometric intersectional stigma scores. Inter-correlations between the respective enacted and anticipated stigma scales are shown in the diagonal. (Note: ^**a**^ **Column showing anticipated stigma scale;** ^**b**^ **Row showing enacted stigma scale ;** ^**c**^ Correlations between intersectional attributes with non-redundant individual attributes; All correlations are statistically significant, p < .01)

Results showed enacted stigma scores were positively correlated with all of the stigma attributions to race, sexual behavior and HIV status as well as all of the combinations of the intersecting attributes (see Fig. 2 upper panel). In addition, the intercorrelations of nonredundant enacted stigma attribute ratings were significant. The same pattern was found for anticipated stigma and the anticipated stigma attributes (see Fig. 2 lower panel). In addition, the enacted and anticipated stigma scales were significantly correlated with each other as were the intercorrelations of their respective attributes and combinations of intersecting attributes (see Fig. 2 diagonal).

### Stigma correlates

The correlations among the enacted stigma scores and correlates are presented in the upper panel of Table 2. Results showed that enacted stigma was positively correlated with stigma distress, depression, loneliness, internalized stigma, medical mistrust, and stigma avoidance behaviors. The enacted stigma attributes and intersectional attributes were all associated with stigma distress, and otherwise showed varied patterns of converging associations with the correlates. Enacted stigma ascribed to HIV was significantly related to all of the correlates except removing medication labels. Stigma ascribed to sexual behavior was more frequently associated with the correlates than was stigma ascribed to race. There was also variability for the correlations among intersectional enacted stigma scores and the correlates, with the intersection of sexual behavior-HIV corelating with all of the correlates except removing medication labels, and the intersection of race-sexual behavior-HIV showing the fewest significant correlations.

**Table 2:** Correlations among enacted and anticipated stigma scales and converging and diverging correlates

Enacted Stigma	Stigma Distress	Depress- ion	Loneliness	Internalized Stigma	Medical Mistrust	Leaves ART at home	Avoids clinic not to be seen	Removes ART labels	Takes ART out of bottles
Enacted Scale score	0.267***	0.408**	0.310**	0.273**	0.375**	0.146**	0.110*	0.099*	0.106*
Race	0.313**	0.048	0.023	0.042	0.118*	0.102*	0.072	0.037	0.096
Sex behavior	0.341**	0.090	0.120*	0.102*	0.083	0.072	0.096	0.073	0.144**
HIV	0.396**	0.205**	0.189**	0.114*	0.148**	0.125**	0.128**	0.085	0.115*
Race-Sex behavior	0.372**	0.072	0.080	0.068	0.107*	0.087	0.082	0.060	0.129**
Race-HIV	0.429**	0.143**	0.121*	0.088	0.143**	0.119*	0.096	0.072	0.125*
Sex behavior-HIV	0.413**	0.153**	0.168**	0.118*	0.106*	0.100*	0.102*	0.083	0.143**
Race-Sex behavior-HIV	0.434**	0.124**	0.128	0.092	0.122*	0.102*	0.091	0.073	0.138**
**Anticipated Stigma**									
Anticipated Scale score	0.301**	0.183**	0.224**	0.230**	1.38**	0.128**	0.114*	0.001	0.037
Race	0.241**	0.021	− 0.005	0.004	0.040	0.008	0.014	− 0.070	0.087
Sex behavior	0.235**	0.023	0.031	0.068	0.070	− 0.016	0.044	0.061	0.163**
HIV	0.348**	0.164**	0.158**	0.139**	0.141**	0.007	0.114	0.027	0.046
Race-Sex behavior	0.262**	0.025	0.020	0.025	0.065	0.002	0.016	− 0.013	0.139**
Race-HIV	0.375**	0.105*	0.086	0.071	0.083	− 0.008	0.062	− 0.033	0.086
Sex behavior-HIV	0.340**	0.101*	0.095	0.112*	0.101	− 0.018	0.082	0.046	0.124**
Race-Sex behavior-HIV	0.353**	0.080	0.069	0.071	0.084	− 0.009	0.051	− 0.005	0.121**

Note: ** p < .01, * p < .05

A similar pattern of results emerged for anticipated stigma, with anticipated stigma associated with all of the correlates except removing medication labels. The attributions for anticipated stigma to race, sex behavior and HIV status correlated with stigma distress. In addition, anticipated stigma ascribed to HIV was significantly related to depression, loneliness, internalized stigma, and medical mistrust. The race-HIV intersection was associated with depression, and sex behavior-HIV was correlated with depression and internalized stigma.

### Stigma and ART adherence

Results for the regression models predicting adherence from enacted stigma and nonredundant individual and intersectional attributes showed that enacted stigma was significantly associated with ART adherence, beta (ß) = − 0.106, t = 2.02, p = .044. There was also a trend toward ascribing stigma experiences to HIV being associated with ART adherence, ß = − 0.111, t = 1.75, p = .080. None of the other individual attributes and intersectional attributes for enacted stigma were significantly associated with ART adherence.

With respect to anticipated stigma, regression models indicated a significant association between the anticipated stigma scale and adherence, ß = − 0.129, t = 2.13, p = .033. Anticipated stigma ascribed to sexual behavior was associated with adherence, ß = -208, t = 2.52, p = .012, and there was a trend toward anticipated stigma ascribed to HIV being associated with adherence, ß = − 0.118, t = 1.79, p = .074. In addition, adherence was predicted by the intersection of race-sexual behavior, ß = 0.162, t = 2.71, p = .007, as well as the intersection of sexual behavior-HIV, ß = 0.111, t = 1.87, p = .062. Finally, the intersectional stigma score for race-sexual behavior-HIV was significantly associated with adherence, ß = 0.136, t = 2.26, p = .024.

## Discussion

The intersectional enacted and anticipated stigma scales demonstrated response variability, internal consistency, and a pattern of associations that suggests construct validity. In addition, the associations between common correlates of stigma differed for the enacted and anticipated intersectional stigma scales, and these associations were consistent with what would be expected to affirm construct validity. It is important to note that the attributes selected for this study (race, same-sex sexual behavior and HIV status) may not have been the most salient stigmatized attributes for participants. A strength of this measurement approach is that it does not assume the assessed attributes are the most salient, but rather the geometric approach allows for isolating attributes of interest to researchers in any given study as well as examining them separately and intersectionally.

With respect to associations between the stigma scales and ART adherence, we observed that the enacted stigma scale was significantly related to ART adherence and there was a trend toward adherence correlating with attributing enacted stigma to HIV; the poorer one’s adherence the more enacted stigma experiences were ascribed to HIV. Thus, while enacted stigma experiences were related to adherence, as is often the case (Kalichman et al., 2020), we did not observe the attributions for stigma to race and sexual behavior, as well as any intersectional stigma score, to be associated with adherence. However, these results are in contrast to those for anticipated stigma, where poorer adherence was associated with greater anticipated stigma scores, and better adherence was associated with ascribing anticipated stigma to sexual behavior, as well as the intersections of race-sexual behavior and race-sexual behavior-HIV. These findings illustrate an advantage to the geometric approach is that it allows for separate assessments of stigma experiences (both enacted and anticipated) from the personal attributes to which one ascribes experiencing stigma. These findings suggest that measures which combine stigma experiences with stigma attributes, such as at the item-level, may yield results that reflect the stigma experiences themselves rather than the attributes to which the experience is assumed to be ascribed. One possible explanation for these results is that while more frequently experienced and anticipated stigma has detrimental impacts on ART adherence, believing one is stigmatized because ‘of who they are’ rather than their having HIV may offer some sense of resilience or other intrapersonal resources that serve to bolster adherence. Longitudinal studies are needed to determine the durability and directions of these associations.

This study represents an initial test of the geometric intersectional HIV stigma scales. The study is limited by relying entirely on self-report measures for tests of construct validity, a particular concern for ART adherence. In addition, the study was limited to Black/African American sexual minority men living with HIV and may not reflect how the scales will function in other populations. Another limitation is that the scales were lengthy in terms of numbers of items and numbers of responses required, which may have been burdensome for participants. Shorter forms of the scale will therefore be tested in future studies. With respect to advantages, this new approach yields scores for enacted and anticipated stigma that are separate from the attributions for these stigma experiences. This is an advantage of the method given the conceptual distinction between experiencing stigma and beliefs about why one experiences stigma. The approach is also powerful in terms of specificity of attributions and combinations of attributions. The enacted and anticipated stigma scales each yielded a total score and six attribution and intersecting attribution scores. Finally, the scale is adaptable to any set of stigma experiences, such as substance use, depression, or obesity stigma, as well as various personal attributes, such as gender, age, and socio-economic status.

Our geometric approach to assessing intersectional stigma demonstrated promising results that warrant further investigation. Of particular importance will be longitudinal studies to test the predictive value of the intersectional stigma scales. For some health behaviors, such as ART adherence, the impact of enacted stigma experiences may occur without association to the attributions people make for having had those experiences. In contrast, the attributes to which individuals ascribe anticipated stigma may influence adherence and these impacts may differ for different combinations of intersecting personal attributes. The level of specificity offered by the geometric measurement approach may prove beneficial in research aiming to refine stigma theories, improve understanding of intersectional stigma, and ameliorate the impacts of stigma on people living with HIV.
